# A Machine Learning Approach to Study Glycosidase Activities from *Bifidobacterium*

**DOI:** 10.3390/microorganisms9051034

**Published:** 2021-05-11

**Authors:** Carlos Sabater, Lorena Ruiz, Abelardo Margolles

**Affiliations:** 1 Department of Microbiology and Biochemistry of Dairy Products, Instituto de Productos Lácteos de Asturias (IPLA), Consejo Superior de Investigaciones Científicas (CSIC), Paseo Río Linares S/N, 33300 Villaviciosa, Asturias, Spain; lorena.ruiz@ipla.csic.es (L.R.); amargolles@ipla.csic.es (A.M.); 2 Instituto de Investigación Sanitaria del Principado de Asturias (ISPA), 33011 Oviedo, Asturias, Spain

**Keywords:** bifidobacteria, glycosidase, prebiotics, machine learning, metagenomics, metagenome-assembled genomes

## Abstract

This study aimed to recover metagenome-assembled genomes (MAGs) from human fecal samples to characterize the glycosidase profiles of *Bifidobacterium* species exposed to different prebiotic oligosaccharides (galacto-oligosaccharides, fructo-oligosaccharides and human milk oligosaccharides, HMOs) as well as high-fiber diets. A total of 1806 MAGs were recovered from 487 infant and adult metagenomes. Unsupervised and supervised classification of glycosidases codified in MAGs using machine-learning algorithms allowed establishing characteristic hydrolytic profiles for *B. adolescentis*, *B. bifidum*, *B. breve*, *B. longum* and *B. pseudocatenulatum*, yielding classification rates above 90%. Glycosidase families GH5 44, GH32, and GH110 were characteristic of *B. bifidum*. The presence or absence of GH1, GH2, GH5 and GH20 was characteristic of *B. adolescentis*, *B. breve* and *B. pseudocatenulatum*, while families GH1 and GH30 were relevant in MAGs from *B. longum*. These characteristic profiles allowed discriminating bifidobacteria regardless of prebiotic exposure. Correlation analysis of glycosidase activities suggests strong associations between glycosidase families comprising HMOs-degrading enzymes, which are often found in MAGs from the same species. Mathematical models here proposed may contribute to a better understanding of the carbohydrate metabolism of some common bifidobacteria species and could be extrapolated to other microorganisms of interest in future studies.

## 1. Introduction

*Bifidobacterium* is one of the most abundant genera of Gram-positive anaerobes that compose the human intestinal microbiota, especially in the early stages of life, during which bifidobacteria may comprise up to 90% of infant gut microbiota [1,2]. It has been proposed that bifidobacteria play a key role in human health, both in infancy and later life stages [3]. Breastfeeding provides infants with a wide variety of natural prebiotic structures, called human milk oligosaccharides (HMOs), which comprise the third most abundant solid component of human milk after lactose and lipids [2]. Remarkably, some HMOs structures are selectively fermented by specific bifidobacterial species typically associated with the breastfed infant gut microbiota and thus are suggested to promote their persistence in the infant gut. Some health benefits associated with *Bifidobacterium* include reducing inflammation, supplementing nutrients through the production of folate and other vitamins, enhancing immune responses to vaccinations, preventing or reducing allergic diseases, and conferring resistance against human pathogens [2,4]. In addition, the probiotic properties of some bifidobacterial strains have been demonstrated and are currently commercialized as probiotic products [5].

Dietary carbohydrates may shape the bifidobacterial pan-genome [3]. Efforts have been made to elucidate glycosidase domains codified in *Bifidobacterium* genomes involved in the hydrolysis of HMOs and galactooligosaccharide (GOS) [2], one of the main oligosaccharide types widely recognized as prebiotics and which is commonly added to infant formula in combination with fructo-oligosaccharides (FOS) to mimic biological functions of HMOs [6]. However, few studies report the recovery of metagenome-assembled genomes (MAGs) of *Bifidobacterium* to study the glycolytic activities of each species. In recent works, Asnicar et al. [7] reported the recovery of 48,181 MAGs, including *Faecalibacterium prausnitzii* or *Bifidobacterium animalis*, while Pasolli et al. [8] reconstructed 154,723 microbial genomes, including *B. adolescentis*, *B. bifidum*, *B. breve*, *B. dentium* and *B. longum* and *Faecalibacterium* species.

On the other hand, it should be considered that microbiome experiments generate large volumes of intricate data, which are often difficult to interpret. For this purpose, several computational workflows involving advanced mathematical modeling techniques have been developed. In this sense, implementing machine learning models comprising several families of powerful pattern-recognition algorithms has proven to be of fundamental interest in the main areas and challenges in microbiome research. Some examples of machine learning applications in basic microbiome research include identification of changes in brain structure associated with diet-dependent changes in gut microbiome populations to gain a better understanding of the gut–brain–microbiota axis [9], identification of trends and patterns characteristic of microbiota-associated gastrointestinal disorders to help with the diagnosis based on different clinical symptoms and biochemical findings [10], design microbiome-targeted therapies and *in silico* prediction of drug–microbiome interactions [11]. In addition, more specific applications of mathematical modeling and machine-learning algorithms have been reported in the field of probiotics and metagenomics. Machine learning has been used to optimize probiotic therapeutics in an artificial human gastrointestinal tract [12] and to assist gut microbiome analysis of sequencing data for identifying patients with specific gastrointestinal diseases [13]. Applications to assess the bioactivity of prebiotic carbohydrates have also been reported. Machine learning models were employed to determine the impact of GOS and FOS on microbial taxa in clinical trials [14] and to simulate colonic fermentation and mechanisms of action in silico of novel prebiotic structures in combination with other computational techniques [15].

To our knowledge, no previous attempts have been made to establish specific glycosidase profiles of recovered MAGs from *Bifidobacterium* species exposed to different types of prebiotic structures. Therefore, this study aimed to develop advanced machine learning models to elucidate complete glycosidase activities encoded in *Bifidobacterium* MAGs recovered from human samples. Characteristic glycosidase profiles of *Bifidobacterium* species were compared, determining the influence of HMOs, GOS, FOS and dietary fiber on their metabolic activities.

## 2. Materials and Methods

### 2.1. Data Collection and Genome Recovery from Metagenomes

To compare metabolic patterns and glycosidic activities of the most representative *Bifidobacterium* species, individual genomes were first recovered from human metagenomes. For this purpose, we collected metagenomic sequencing data obtained from two of the most widely known reference repositories: Sequence Read Archive (SRA) from the National Center for Biotechnology Information (NCBI) and MG-RAST metagenomics analysis server. Among all datasets available, two studies reported shotgun metagenomic sequencing of human fecal samples after regular prebiotic consumption or fiber-based dietary interventions [16,17]. The first study comprises 447 paired-end metagenomic sequences from 60 infants (0–8 months of age; 31 females and 29 males) following different diets (breastfed, whole-milk-fed, and infant formula-fed). Infant formulas described were supplemented with different types of prebiotic oligosaccharides: GOS and FOS. In addition, HMOs may play a relevant role in breastfed individuals. These samples were deposited under BioProject identity PRJNA473126, sample accession codes SAMN09259835–SAMN09260236 [16]. On the other hand, the second study consisted of 40 paired-end metagenomic sequences from 39 healthy adults (33 women, 6 men) following two 3 day dietary interventions where they consumed either barley kernel-based bread or white wheat flour bread. These samples were deposited under MG-RAST identity mgp13068, MG-RAST ID of sequences mgm4624578.3–mgm4624657.3 [17].

Sequences had been already quality-filtered, trimmed and decontaminated by the authors. The first step in our data-analysis pipeline involved concatenation of paired-end sequences and metagenome assembly using MEGAHIT v.1.2.9 [18]. Default settings were used for assembly commands with the exception of maximum *k-mer* size, set at 127, generating a series of *k-mers* with different length, which are shorter than entire reads (*k*-21, *k*-31, *k*-41, k-51, *k*-61, *k*-71, *k*-81, *k*-91, *k*-101, *k*-111, *k*-121, *k*-127). Bowtie2 v.2.3.5.1 [19] was run on MEGAHIT results to map the reads against the assembly. Bam files generated were sorted and indexed. Then, assembled contigs ≥1.5 kilobases were grouped and assigned to individual genomes in the binning step performed using MetaBAT2 v.2.2.15 default options [20]. Quality control of the obtained bins was assessed using CheckM v.1.1.3 lineage-specific workflow, the recommended workflow for assessing the completeness and contamination of genome bins [21]. MAGs showing completeness higher than 50% and contamination lower than 5% were selected according to Asnicar et al. [7]. Finally, taxonomic identification of MAGs was performed following Kraken2 standard workflow [22].

Once individual genomes were assembled and identified, glycosidase functional domains of MAGs were annotated following the “run_dbcan” pipeline developed by Zhang et al. [23], which maps the samples against the carbohydrate-active enzymes database (CAZy, http://www.cazy.org/ last accessed: 1 March 2021). HMMER software for biosequence analysis using profile hidden Markov models (HMMs) [24] was integrated into the standard workflow, allowing functional domain annotation and glycosidase identification based on the Pfam database, a widely used resource for identifying functional domains that occur within proteins and the classification of protein sequences into families [25]. To ensure the quality of glycosidase identification, only glycosidase domains showing coverage values higher than 0.95 were selected for further analysis.

### 2.2. Machine Learning Modeling to Elucidate Glycosidase Patterns

Glycosidase functional domains identified in MAGs were grouped into CAZy glycosidase families. To compare metabolic profiles of MAGs recovered, glycosidase profiles were expressed as binary data, including presence (value = 1) and absence (value = 0) of glycosidase domains from the same CAZy family in each MAG. According to different criteria, these data were used as input for different machine learning models, powerful pattern-recognition algorithms that allow accurate classification of samples from their biological origin [26,27]. Our machine-learning data analysis strategy involved three main steps: (i) unsupervised distribution of glycosidase profiles of MAGs according to both *Bifidobacterium* species (*B. adolescentis*, *B. bifidum*, *B. breve*, *B. catenulatum*, *B. dentium*, *B. longum*, *B. pseudocatenulatum*, *B. scardovii*) and diet type (breastfed, breastfed + GOS, breastfed + GOS + FOS, infant formula-fed, infant formula-fed + FOS, infant formula-fed + GOS, infant formula-fed + GOS + FOS and whole-milk-fed infants as well as fiber-rich diets in adults), (ii) supervised classification of MAGs to accurately elucidate characteristic glycosidic patterns of the main bifidobacteria identified (*B. adolescentis*, *B. bifidum*, *B. breve*, *B. longum*, *B. pseudocatenulatum*), (iii) association study between glycosidase families found in MAGs. It should be noted that *B. catenulatum*, *B. dentium* and *B. scardovii* were not considered for supervised classification in step (ii) due to the low number of MAGs recovered from these species, insufficient to train supervised models.

Concerning the first step, the unsupervised distribution of MAG glycosidic activities was investigated through artificial neural network-based principal component analysis (PCA). This model was developed to reconstruct experimental data (i.e., presence/absence of functional domains from a specific CAZy family) by the conventional PCA method in combination with artificial neural networks (ANNs) [26,28]. ANNs are among the most popular families of machine learning models that allow modeling complex and highly nonlinear processes. These complex algorithms are formed by an input layer (i.e., principal components from PCA), an output layer (i.e., reconstructed glycosidase profiles of each MAG) and several “neurons” or nodes organized in a hidden layer (i.e., 20 neurons in this study), connected through mathematical functions. The activation function, a transformation applied to the input spectra to determine whether the information that the neuron is receiving is relevant or not, was a hyperbolic tangent activation function (tanh). Weight decay value, a regularization technique used to avoid over-fitting based on the multiplication of the sum of squares of ANN weights by a smaller number, was 0.001. ANN-based PCA was computed using the “pcaMethods” R package v1.78.0 [28].

The second data analysis step involves a supervised classification of MAGs to elucidate characteristic glycosidase profiles of several *Bifidobacterium* species in an accurate and reproducible way. With this aim, three different supervised machine learning models were compared: ANN, random forest (RF) and generalized linear model elastic-net (glmnet). Similar to the first step, ANN was formed by an input layer (i.e., presence/absence of functional domains from a specific CAZy family), an output layer (i.e., *Bifidobacterium* species) and 5 neurons organized in one hidden layer, where each neuron in a layer was connected with each neuron in the next layer through a weighted connection. The activation function was logistic, and the weight decay value was 0.1. In contrast, RF is based on multiple decision trees (i.e., 500 in this study), outputting the different classes (i.e., bifidobacteria species), and each node is split using the best among a subset of predictors (i.e., presence/absence of functional domains from a specific CAZy family) randomly chosen (i.e., 9 variables tried at each split in this study). Then, RF averages the results from each tree to get a more accurate and stable prediction. The glmnet model involves a linear regression model that could be generalized (i.e., the response variable may follow different distributions than the normal distribution). Regularization methods are used to reduce possible overfitting of generalized linear models and reduce the prediction error variance. Elastic-net regularization is based on two parameters, alpha and lambda. Alpha (comprised between 0 and 1, i.e., 0.4 in this study) is used to optimize the model, and it indicates the combination of two different regularization techniques, L1 and L2. All these methods try to penalize the Beta coefficients (i.e., 0.02 in this case) of the regression for obtaining the important variables. Lambda is the regularization parameter or penalty coefficient and allows adjusting the prediction error. ANN, RF and glmnet models were computed using the nnet v7.3.12, random forest v4.6.14 and glmnet v3.0.2 R packages [29,30,31].

The last data analysis step involved a graphical correlation network between glycosidase families present in MAGs, computed using ccrepe v1.22.0 and qgraph v1.6.5 R packages [32,33].

All mathematical models were computed on R v3.6.2.

## 3. Results

### 3.1. Recovery of Metagenome-Assembled Genomes (MAGs)

Characteristic glycosidase profiles of several *Bifidobacterium* species present in the gastrointestinal tract of both healthy infants and adults were investigated through a survey of MAGs recovered from gut metagenomic datasets. To this end, shotgun metagenomic data have been collected from the main sequence repositories [16,17]. Sequences were assembled into contigs and MAGs, and annotation of glycosidase functional domains and families codified in individual genomes was performed using the CAZy database [23]. A total of 1806 MAGs representatives of 177 species and strains were recovered from 487 metagenomes, comprising 1339 MAGs from 447 metagenomes from 60 infants, as well as 467 MAGs from 40 metagenomes from 39 adults (Supplementary Materials Table S1). *Escherichia coli* (n = 140), *Faecalibacterium prausnitzii* (n = 116), and *Ruminococcus gnavus* (n = 114) showed the highest overall number of MAGs recovered. Asnicar et al. [7] and Pasolli et al. [8] recovered MAGs from *F. prausnitzii* and *R. gnavus* showing the relevance of these species in large cohorts of participants. *Escherichia*, *Faecalibacterium* and *Ruminococcus* were also found in infant metagenomes using assembly-free methods [16].

In general, bifidobacterial species were among the most frequently identified MAGs species, including *B. longum* (n = 70), *B. bifidum* (n = 39), *B. breve* (n = 38) and *B. pseudocatenulatum* (n = 35). However, a low number of MAGs were recovered from other bifidobacteria species, such as *B. adolescentis* (n = 17), *B. dentium* (n = 2), *B. catenulatum* (n = 1) and *B. scardovii* (n = 1). Previous studies also reported MAG recovery from *B. adolescentis*, *B. bifidum*, *B. breve*, *B. dentium* and *B. longum* in fecal metagenomes from human donors following westernized and non-westernized lifestyles [7,8], highlighting the relevance of these species in metagenomes from different individuals (infants and adults) exposed to specific types of diets, including variable prebiotic consumption. Moreover, gene families and metabolic pathways of several species belonging to Bifidobacteriaceae were reported by previous authors in the assembly free analysis of the infant metagenomes [16], in agreement with our assembly results.

Other species and strains showing a high number of MAGs recovered were *Collinsella aerofaciens* (n = 71), *Veillonella parvula* (n = 62), *Akkermansia muciniphila* (n = 59), Lachnospiraceae *bacterium* strain GAM79 (n = 50) and *Ruthenibacterium lactatiformans* (n = 46). MAGs from *A. muciniphila* were also recovered from Pasolli et al. [8] in westernized and non-westernized cohorts in agreement with our results. It has been reported that metabolic pathways of *Collinsella*, *Veillonella parvula* and *Akkermansia muciniphila* played a relevant role in infant microbiota [16], in agreement with the high number of MAGs recovered from these clades.

It should be noted that some MAGs were identified at strain level, including *Blautia* sp. SC05B48 (n = 38), *Ruminococcus* sp. JE7A12 (n = 32), *Lachnoclostridium* sp. YL32 (n = 25), *Longibaculum* sp. KGMB06250 (n = 25), *Caproiciproducens* sp. NJN-50 (n = 19), *Blautia* sp. N6H1-15 (n = 16), Lachnospiraceae bacterium Choco86 (n = 16), Erysipelotrichaceae bacterium GAM147 (n = 15), *Streptococcus* sp. HSISS3 (n = 11) and *Enterococcus* sp. HSIEG1 (n = 8). Interestingly, metabolic pathways of Enterobacteriaceae and Lachnospiraceae families as well as *Blautia* species had been also identified in infant metagenomes using assembly-free methods [16].

Furthermore, up to 11 MAGs could be recovered from *Lacticaseibacillus rhamnosus,* although few MAGs were obtained from other lactobacilli species. On the other hand, *Clostridium bolteae* (n = 36) was the most frequent *Clostridium* species, while a low number of MAGs could be obtained from other genera like *Bacillus*, *Staphylococcus* and *Streptococcus*. Interestingly, *Clostridium* genes were also identified in the assembly-free analysis of infant metagenomes [16].

Concerning the sample origin of each MAG, most MAGs were recovered from formula-fed infants (n = 638), adults following fiber-rich dietary interventions (n = 467) and infant formula + GOS-fed infants (n = 281). Similarly, most bifidobacteria MAGs were obtained from formula-fed infants (n = 64), infant formula + GOS-fed infants (n = 52) and breastfed infants (n = 31), while only 11 bifidobacteria MAGs could be obtained from adult participants (Supplementary Materials Table S2). Among these samples, most MAGs from *B. adolescentis* were recovered from adults following fiber-rich dietary interventions (n = 8), while most MAGs from *B. longum* and *B. pseudocatenulatum* were recovered from infant formula-fed infants (n = 32 and n = 13, respectively). Similarly, most MAGs from *B. bifidum* and *B. breve* were recovered from breastfed (n = 11) and infant formula + GOS-fed infants (n = 16), respectively.

To deepen the metabolic potential of the MAGs species and strains obtained in this study and their variation with prebiotics consumption, glycosidase activities codified in MAGs were annotated, finding up to 80,028 functional domains belonging to 54 CAZy families of interest. These CAZy families were chosen based on their potential ability to metabolize the most common types of prebiotics (α- and β-GOS and FOS commonly added to infant formula), as well as HMOs. It must be considered that these oligosaccharide structures were present in the diet of most individuals considered in this study [16]. Specific glycosidase activities aimed at hydrolyzing these substrates were selected to find characteristic metabolic profiles of the *Bifidobacterium* genus depending on prebiotic consumption. Moreover, enzymatic profiles of prebiotic-exposed bifidobacterial MAGs were compared to those of bifidobacteria found in infants fed by non-supplemented formulas and adults exposed to high-fiber dietary interventions (and not specific prebiotic structures). Therefore, functional domains from CAZy families CBM32, CBM40, GH1-5, GH16, GH20, GH29, GH30-33, GH35-36, GH39, GH42, GH58-59, GH68, GH95, GH97, GH109-110, GH139, GH141, GH147 and GH151 involving α- and β-galactosidases, β-fructosyltransferases and β- fructofuranosidases, fucosidases, hexosaminidase and sialidases as well as other fiber-degrading activities were selected (Supplementary Materials Table S3). Most metabolic activities reported during the assembly-free functional analysis of infant microbiota were limited to routes related to amino acid synthesis and degradation and vitamin synthesis pathways [16]. In contrast, the present study deepens the characterization of carbohydrate-degrading enzymes present in these species, providing an extensive comparison of CAZy families found in *Bifidobacterium* species and their ability to metabolize specific prebiotic structures.

### 3.2. Unsupervised Analysis to Study Glycosidase Distribution in Metagenome-Assembled Genomes (MAGs)

**Figure 1:**
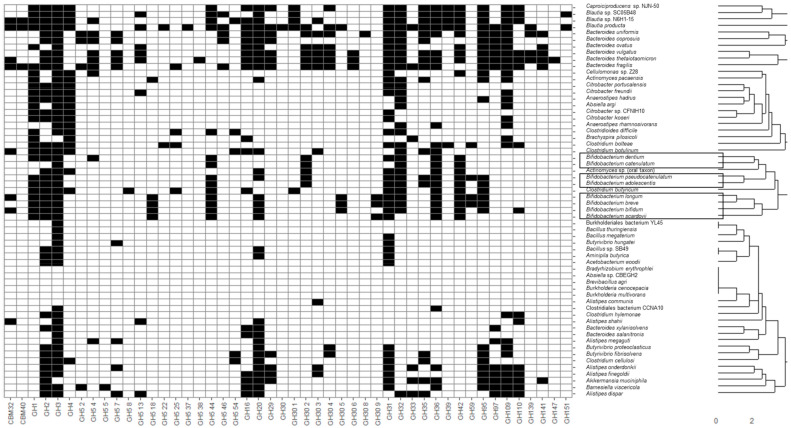

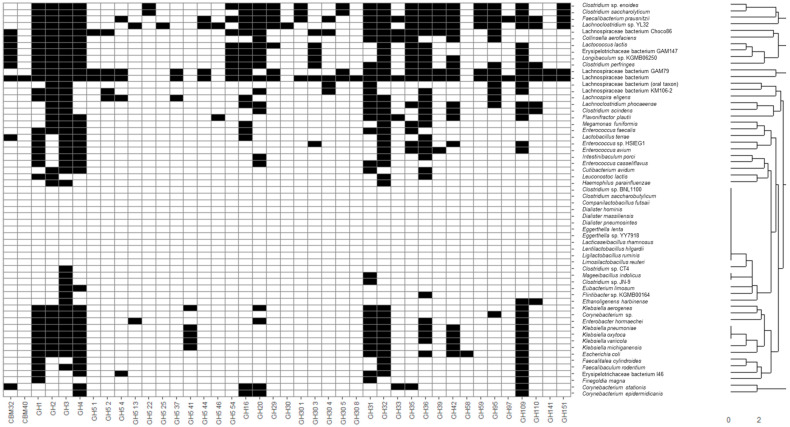

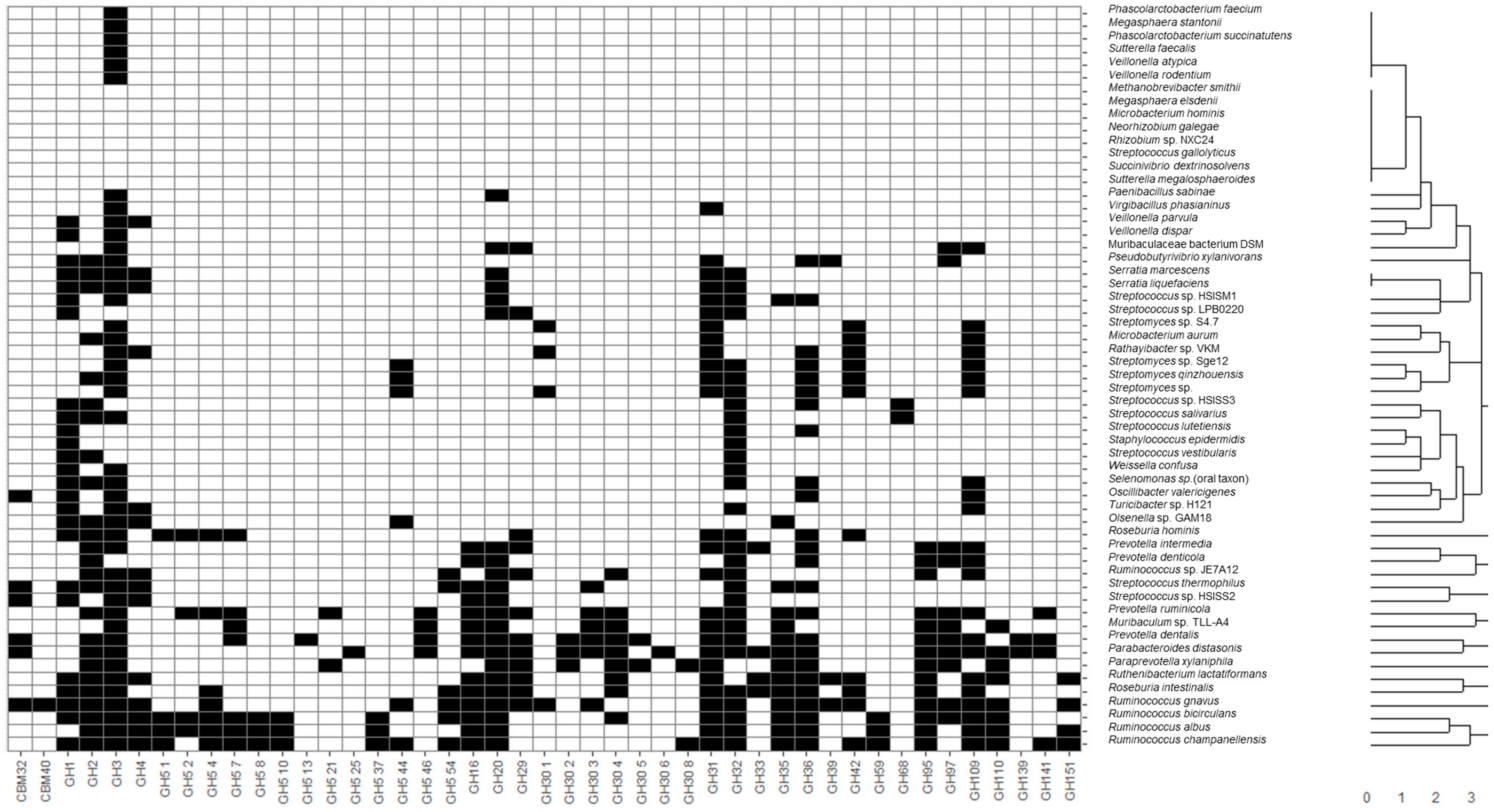
Heatmap showing the presence of different glycosidases (indicated as black cells) in metagenome-assembled genomes (MAGs). Specifically, glycosidases capable of degrading galacto- and fructo-oligosaccharides (GOS and FOS), as well as human milk oligosaccharides (HMOs), are illustrated. Black and white cells indicate the presence and absence of a specific CAZy family, respectively. Codes corresponding to the CAZy family of each enzyme were assigned. MAGs from *Bifidobacterium* are highlighted in black. (top) Glycosidase functional domains corresponding to CAZy families GH5 1, GH5 10, GH5 21, GH5 41, GH58 and GH68 were not identified in MAGs from the species shown in this section of the heatmap (vertical axis) and are not illustrated. (middle) Glycosidase functional domains corresponding to CAZy families GH5 5, GH5 7, GH5 8, GH5 10, GH5 18, GH5 21, GH5 38, GH30 2, GH30 6, GH30 9, GH68, GH139 and GH147 were not identified in MAGs from the species shown in this section of the heatmap (vertical axis) and are not illustrated. (bottom) Glycosidase functional domains corresponding to CAZy families GH5 5, GH5 18, GH5 22, GH5 38, GH5 41, GH30, GH30 9, GH58 and GH147 were not identified in MAGs from the species shown in this section of the heatmap (vertical axis) and are not illustrated.

Recovered MAGs from all identified microbial species were clustered considering the presence of CAZy families of interest described in the previous section through hierarchical clustering considering a Euclidean distance metric (Figure 1). Moreover, heatmaps illustrating the presence and absence of functional domains were generated. In this sense, applications of heatmaps to represent the presence or absence of microbial taxa and genes have been extensively reported in the literature. Some recent examples include the usage of heatmaps to elucidate the presence of coincident genes across microbial genomes [34] and coincident genera in human microbiota samples [35]. In the present study, most species belonging to the same genus were clustered together, highlighting common metabolic patterns. In general, the widest number of glycosidase activities codified in MAGs was observed for the following genera: *Bacteroides*, *Blautia*, *Caproiciproducens*, *Clostridium*, *Longibaculum*, *Paraprevotella*, *Prevotella*, *Roseburia*, *Ruminococcus*, *Ruthenibacterium*, *Muribaculum*, as well as several species belonging to Lachnospiraceae and Erysipelotrichaceae families (Figure 1). It should be noted that *Bacteroides,* belonging to Bacteroidetes phyla, is one of the most relevant clades in the microbiota of adult individuals due to particular metabolic capabilities that allow *Bacteroides* to use a wide range of complex carbohydrates [36]. Other Bacteroidetes, such as *Paraprevotella*, *Prevotella*, *Muribaculum*, may play a similar role based on their glycosidase profiles. In addition, *Roseburia* comprises another relevant genus of the Firmicutes phyla known to metabolize dietary polysaccharides [17]. Other Firmicutes that exhibited a wide range of prebiotic-degrading enzymes include *Blautia*, *Caproiciproducens*, *Clostridium*, *Longibaculum*, *Ruminococcus* and *Ruthenibacterium.* It has been reported that the healthy gut microbiome is composed predominantly of the phyla Firmicutes and Bacteroidetes [37], and our cluster analysis highlights the prevalent role of the glycosidic metabolic activities of these two phyla, showing a wider variety of glycosidases able to hydrolyze some of the most common types of prebiotics.

Glycosidase activities from these bacteria corresponded mainly to the CBM32, CBM40, GH1-5, GH31-42, GH95-110 CAZy families comprising most enzyme classes included in this study, such as α- and β-galactosidases, fucosidases, fructosidases, hexosaminidases and sialidases. On the contrary, other species not characteristic of a healthy infant microbiota, according to Baumann-Dudenhoeffer et al. [16] and Hill et al. [38], lack several GH5 and GH30 subgroups comprising hexosaminidases and fucosidases, indicating a limited potential ability to metabolize HMOs.

A wide number of glycosidase activities were also found for *Bifidobacterium*, similar to those of the above-mentioned non-bifidobacteria species with high metabolic capacities, belonging to Firmicutes and Bacteroidetes. Moreover, some glycosidases were characteristic of bifidobacteria (i.e., not present in most MAGs species recovered): GH30 5 fucosidases were present in *B. bifidum*, *B. breve* and *B. longum,* as well as other non-bifidobacteria species like *B. producta*, *C.* sp. *enoides*, *C*. *saccharolyticum*, *P*. *dentalis* and *P*. *xylaniphila*, and *L*. *bacterium* strain GAM79. Similarly, GH59 β-galactosidases were characteristic of *B*. *breve*, *B*. *longum* and *B*. *pseudocatenulatum*, but were also present in *C*. *bolteae*, *C.* sp. *enoides*, *C*. *saccharolyticum*, *F. prausnitzii*, *R*. *albus*, *R*. *bicirculans* and *R*. *champanellensis*, and strains *L.* sp. YL32 and *L*. *bacterium* GAM79. As it can be seen, some characteristic glycosidases from bifidobacterial were also found in the *Clostridium* and *Ruminococcus* genera and novel strains from Lachnospiraceae, highlighting metabolic similarities between these species and a higher potential to degrade HMOs and mucins when compared to other microorganisms.

Other non- bifidobacteria species showed a limited number of CAZy families of interest, more limited than those observed for bifidobacteria. In this sense, only GH3 and GH31, comprising hexosaminidases and α-galactosidases, were found in *Mageeibacillus indolicus,* while GH3 was the only relevant domain analyzed in MAGs from *Phascolarctobacterium* and *Megasphaera* genera. The presence of α-galactosidases in these species could be of interest to metabolize α-GOS commonly added to infant formula due to their potential prebiotic effect [6]. Interestingly, GH30 3 family involving fucosidases was the only relevant domain determined in *Alistipes communis* MAGs. It should be considered that fucosidases have been described in species from the genus *Alistipes* [39].

Main glycosidase activities for several species and strains presented in this work were previously reported in adult metagenomes by other authors involving xylan 1,4-beta-xylosidase, glucan endo-1,3-beta-D-glucosidase, glucan 1,6-alpha-glucosidase, licheninase, and cellulase [17]. However, no attempts to elucidate glycosidase profiles of common prebiotic structures like GOS and FOS were made. Therefore, the results herein presented provide complementary information to those already reported in the bibliography and may provide a foundational basis to estimate which prebiotic structures could be more fermentable by a given species.

**Figure 2:**
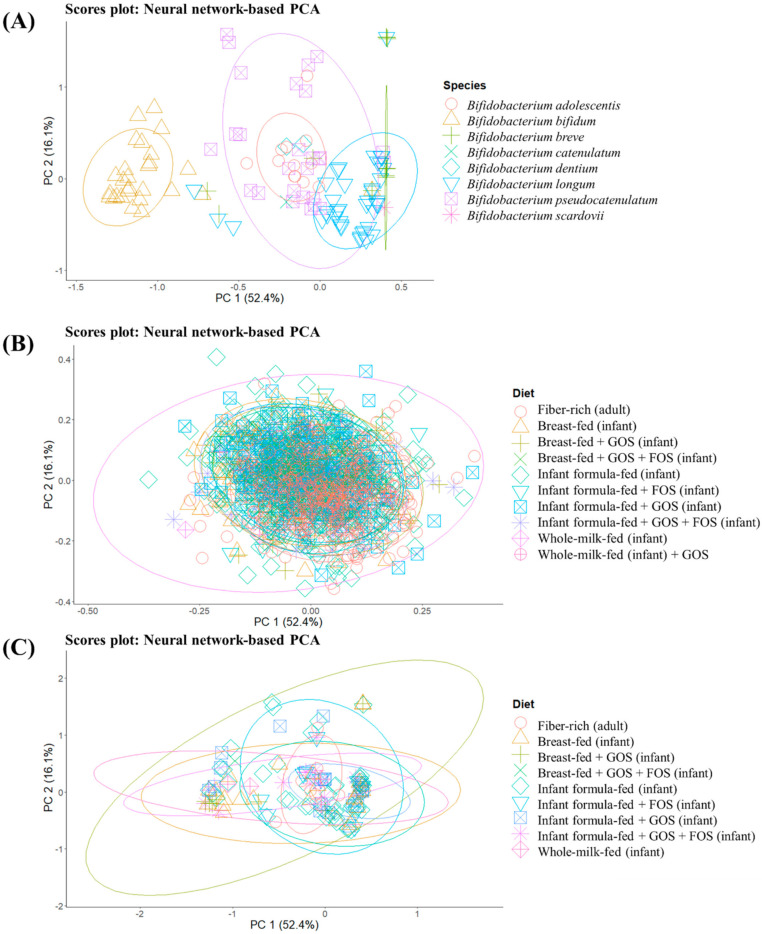
Artificial neural network-based principal component analysis (PCA) of glycosidase activities encoded in metagenome-assembled genomes (MAGs). MAG distribution is illustrated according to two different criteria: characteristic profiles of MAG-recovered *Bifidobacterium* species (**A**), differences in MAGs from all identified species (including not only bifidobacteria) (**B**) and only bifidobacteria species (**C**) depending on the diet. GOS: galacto-oligosaccharides, FOS: fructo-oligosaccharides.

To get a general overview of glycosidase activities codified in different MAGs obtained from groups consuming different prebiotic structures, unsupervised sample distribution was evaluated by the ANN-based PCA model (Figure 2). Specifically, differences between MAGs from different *Bifidobacterium* species (Figure 2A) as well as glycosidase profiles of MAGs from all identified species (and not just *Bifidobacterium*, Supplementary Materials Table S1) according to the host diet (Figure 2B,C), are illustrated. The implementation of an ANN allows describing as much variance as possible, and the cumulative percentage of variance explained by the first five components was 89.2%, which could not be achieved by conventional PCA. Therefore, this kind of mathematical model could be especially suitable to find patterns in biological samples, which may yield sparse and heterogeneous data [26,27]. *B. bifidum* showed a characteristic glycosidic profile different from the rest of bifidobacteria (*B. adolescentis*, *B. breve*, *B. catenulatum*, *B. dentium*, *B. longum*, *B. pseudocatenulatum*, *B. scardovii*) (Figure 2A). This could be related to the potential ability of this species to degrade mucins [40]. In addition, glycosidase patterns of *B. longum* were distinguished from those of *B. adolescentis*, considering the absence of overlap in normal ellipses and the percentages of variance explained.

When interpreting these differences, it should be considered that metabolic activities of *B. bifidum* and *B. longum* subsp. *infantis* are tailored toward HMOs degradation, while other bifidobacteria do not encode the same HMOs-specific glycosidases and can degrade only limited HMOs [41]. In contrast, no characteristic pattern could be elucidated when grouping MAGs from bifidobacteria and other species identified (Figure 2B,C) according to the type of diet (breastfed, breastfed + GOS, breastfed + GOS + FOS, infant formula-fed, infant formula-fed + FOS, infant formula-fed + GOS, infant formula-fed + GOS + FOS and whole-milk-fed infants as well as fiber-rich diets in adults).

These results indicate the existence of different glycosidase profiles between *Bifidobacterium* species regardless of prebiotic exposure.

### 3.3. Supervised Classification to Establish Characteristic Glycosidase Profiles of Bifidobacterium Species

**Figure 3:**
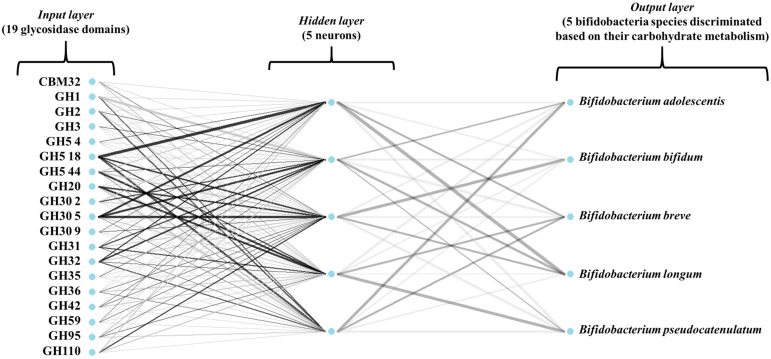
Artificial neural network (ANN) architecture computed to classify metagenome-assembled genomes (MAGs) of *B. adolescentis*, *B. bifidum*, *B. breve*, *B. longum* and *B. pseudocatenulatum* based on their glycosidase activities. Weights are color-coded by sign (black +, gray -); thickness is in proportion to magnitude.

To deepen the study of characteristic glycosidases found in *Bifidobacterium* MAGs, several supervised classification algorithms were compared, allowing establishing more robust patterns than the ones suggested by unsupervised projection. The number of MAGs recovered from *B. catenulatum*, *B. dentium* and *B. scardovii* was not enough to train supervised algorithms (Supplementary Materials Table S1), so these models were focused on *B. adolescentis*, *B. bifidum*, *B. breve*, *B. longum*, *B. pseudocatenulatum*. ANN, RF and glmnet were trained on 70% of MAGs from each *Bifidobacterium* species and tested on 30% new samples to ensure the reproducibility of the model. Figure 3 illustrates the architecture of the ANN model used in this study. In addition, all models were 10-fold cross-validated. To assess model performance, several estimators were calculated (Supplementary Materials Table S4). The number of correctly classified MAGs during train and test phases was 97.8 and 91.7% for all models, while cross-validation accuracies for ANN, RF and glmnet were 96.4, 96.4 and 97.1%, respectively. Cross-validation kappa values, a more robust measure of accuracy that takes into account the possibility of correct classification by chance, for ANN, RF and glmnet, were 95.2, 96.2 and 96.2%, respectively. Similarly, kappa values obtained during the test phases were 89.2, 89.1 and 89.1% for ANN, RF and glmnet. A comparative account of the models was performed (Supplementary Materials Figure S1), highlighting the absence of statistically significant differences (*p* > 0.05) accuracies and kappa values calculated from the resampling distributions of the three algorithms. Additional estimators calculated include model sensitivity, specificity, precision, recall, F1, and balanced accuracy during the test phase on new samples.

Sensitivity, defined as the coefficient of the number of true-positive results by the total number of positives (including false-positives), recall, defined as the coefficient of true positives between relevant elements (i.e., MAGs from the same bifidobacteria species), and F1 coefficient, which combines precision and recall in one metric, showed lower values for *B. adolescentis* (ranging from 0.63–0.83) than the rest of bifidobacteria in all models. This fact indicates a lower classification rate for *B. adolescentis* than the rest of *Bifidobacterium* species, revealing the absence of a unique glycosidase pattern for this species. As a consequence, characteristic glycosidases of *B. adolescentis* are also characteristic of other bifidobacteria. However, specificity calculated by dividing the number of true-negative results by the total number of negatives (including false negatives) presented high values for all species, above 0.95 (Supplementary Materials Table S4). Interestingly, precision, defined as the percentage of the model’s positive predictions that are accurate, showed the lowest values for *B. pseudocatenulatum* (0.82 for the three models). As a consequence, *B. adolescentis* and *B*. *pseudocatenulatum* exhibited the lowest balanced accuracies (0.81–0.86 and 0.93, respectively), defined as the sum of sensitivity and specificity divided by two.

These results could be attributed to minor differences existing in the glycosidase profiles of *B. adolescentis* and *B. pseudocatenulatum* recovered from infant or adult metagenomes and metabolic similarities between these two species, leading to misclassification of certain MAGs. Indeed, these two species belong to the same phylogenetic group [42]. Machine learning trained MAG glycosidase data showed similar performance metrics than previous models trained on carbohydrate spectral data, highlighting the suitability of this mathematical approach to elucidate complex patterns within the field of probiotics and prebiotics [27].

When interpreting these models, it should be considered that RF, glmnet and MLP are computed differently, although they yielded similar results, highlighting the characteristic activities of each *Bifidobacterium* species. These profiles were elucidated by complementary approaches, and the results from predictive algorithms reinforce each other. The unsupervised method could not properly discriminate between all *Bifidobacterium* species showing a lower performance (low percentages of variance explained by the first components). In contrast, supervised ANN, RF and MLP showed high classification accuracies, above 90% when tested on new samples. This fact may be attributed to the subtle differences existing between glycosidase domains found in MAGs that could not be properly explained by PCA-like methods. Therefore, advanced supervised pattern recognition methods are needed to elucidate the metabolic profiles of bifidobacteria.

To determine the most relevant glycosidase activities in the characteristic profile of *Bifidobacterium* species, a variable importance analysis of each model was carried out (Supplementary Materials Tables S5–S7). The most dominant glycosidase family from *B. adolescentis* was GH2 (involving β-galactosidases). The absence of GH20 (comprising hexosaminidases) was also characteristic of this species, although *B. adolescentis* shared the rest of its characteristic glycosidase domains with other *Bifidobacterium* species, leading to a lower classification rate (Supplementary Materials Table S4). Pokusaeva et al. [43] suggested that *B. adolescentis* is incapable of properly degrading HMOs, agreeing with our findings.

Relevant families in *B. bifidum* profiles include GH5 44, GH32, and GH110 (hexosaminidases, fructosidases, fructosyl transferases and α-galactosidases). It should be noted that the GH110 CAZy family was not found in MAGs from other bifidobacteria (Figure 1), indicating a different hydrolytic profile for *B. bifidum* that may explain metabolic differences observed in PCA analysis (Figure 2A). The role of hexosaminidases like β-N-acetylglucosaminidases on HMOs metabolism has been described by Sakanaka et al. [2]. Specifically, the presence of hexosaminidases reported in *B. bifidum, B. breve* and *B. longum* subsp. *infantis* genomes has been reported. Hexosaminidases from *B. bifidum*, showing high importance coefficients in our machine learning models, could be highly active on HMOs like lacto-N-triose (LNTri), while those from other species like *B. breve* could be active on lacto-N-tetraose (LNT) and lacto-N-neotetraose (LNnT). It has also been reported that *B. bifidum* shows a high HMOs assimilation ability that may contribute to its characteristic glycosidase profile that favors its persistence in the breastfed infant gut [2,43]. In fact, previous studies indicate that *B. bifidum* degrades some HMOs structures more rapidly than lactose [43]. After host glycans and HMOs degradation, mono– or disaccharides released are consumed by other species metabolically dependent on these simple sugars [4,44].

Glycosidase profiles of *B. breve* were characterized by GH1 and GH5 18 families (comprising β-galactosidases and hexosaminidases hydrolyzing mainly LNT and LNnT in contrast to those from *B. bifidum*), and those from *B. longum* showed high importance coefficients for β-galactosidases and fucosidases from GH1 and GH30 5 families. Finally, the presence of the glycosidase families, GH5 44 and GH20 comprising hexosaminidases and the absence of GH5 18 were characteristic traits from *B. pseudocatenulatum*. These enzymes may contribute to the metabolism of lacto-N-biose I (LNB), according to previous authors [2,45].

We have demonstrated that it is possible to get highly accurate classifications of glycosidase activities from some of the most common *Bifidobacterium* species based on several mathematical approaches. Specifically, ANN, RF and glmnet exhibited high-performance metrics, indicating a high predictive power. As explained, characteristic glycosidase profiles of each bifidobacteria have been elucidated based on the importance coefficients from the three machine learning models. These models could be generalized and applied to new genomes from bifidobacteria and related microorganisms in future studies.

Concerning the main limitations of this method, it is possible that some glycosidase domains of interest may be lost during MAG assembly. However, the study of bifidobacteria MAGs allows comparing metabolic profiles of *Bifidobacterium* associated with specific groups of individuals (i.e., following dietary interventions of interest) and assessing the metabolic complementarity between bifidobacteria and non-bifidobacteria species found in the same participant.

### 3.4. Correlation Networks to Elucidate Glycosidase Activities Commonly Associated

**Figure 4:**
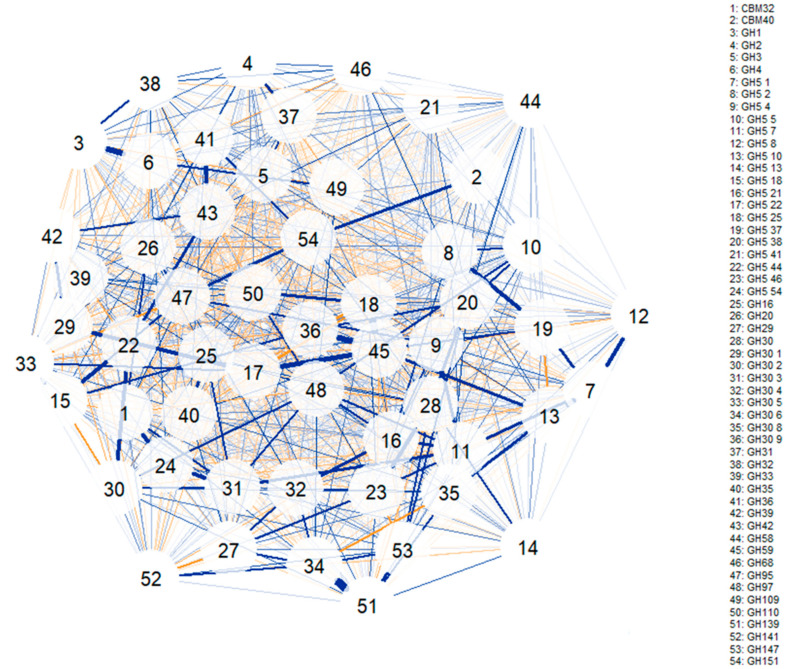
Correlation network illustrating positive and negative associations between glycosidase activities capable of degrading galacto- and fructo-oligosaccharides (GOS and FOS) as well as human milk oligosaccharides (HMOs), encoded in metagenome-assembled genomes (MAGs) from all species identified (bifidobacteria or not). Blue lines indicate positive associations, while saffron lines suggest negative associations. Line thickness is in proportion to magnitude. Specific activities included in each glycosidase family can be found in Supplementary Materials Table S3.

**Figure 5:**
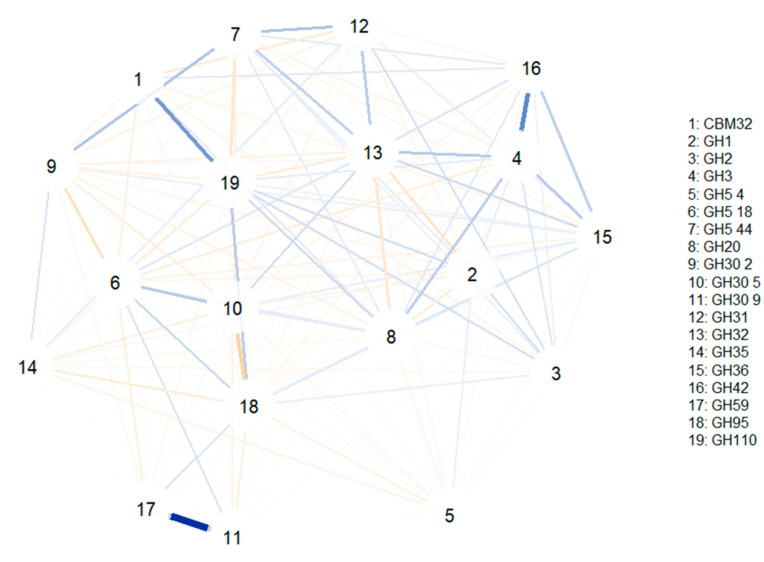
Correlation network illustrating positive and negative associations between glycosidase activities capable of degrading galacto- and fructo-oligosaccharides (GOS and FOS) as well as human milk oligosaccharides (HMOs), encoded in metagenome-assembled genomes (MAGs) from *Bifidobacterium*. Blue lines indicate positive associations, while saffron lines suggest negative associations. Line thickness is in proportion to magnitude. Specific activities included in each glycosidase family can be found in Supplementary Materials Table S3.

To study the associations between different glycosidase activities (i.e., which glycosidase families are usually encoded together in the same MAG), correlation network models were computed (Figure 4 and Figure 5). The first correlation model (Figure 4) was built using glycosidase activities from all species identified (bifidobacteria or not). Some of the strongest correlations observed include the positive associations between GH1 and GH4 and between GH36 and GH42 (involving α- and β-galactosidases). Furthermore, GH139 was positively associated with GH30 6 and GH147 families (involving β-galactosidases and fucosidases), and the GH151 family was correlated to CBM40 and GH97 (involving α-galactosidases, fucosidases and sialidases). In this sense, GH5 44 was associated with GH30 1, GH16, GH5 18 and GH30 2 families and GH59 was positively correlated to GH30 9 and GH5 22 families (comprising β-galactosidases, fucosidases and hexosaminidases). Similarly, several CAZy families involving hexosaminidases showed positive correlations: GH5 37 was associated with GH5 2, GH5 4 and GH5 1, while GH5 1 was associated with GH5 8, and GH5 22 was associated with GH5 9. It should be noted that all negative correlations were weaker than the positive ones, and no relevant associations were observed.

A second correlation network was computed to investigate glycosidase associations only in bifidobacteria MAGs (Figure 5). GH30 9 and GH59 families involving fucosidase and β-galactosidase activities were positively associated, while a positive association was found between CBM32 and GH110 (comprising sialidases and α-galactosidases characteristic of *B. bifidum*). In addition, GH3 was correlated to GH4; these two CAZy families comprising hexosaminidases and β-galactosidases. Similar to the previous study, no relevant negative correlations were observed.

As previously explained, fucosidases and β-galactosidases were strongly correlated not only in bifidobacteria but in all MAGs analyzed. It should be considered that these two enzyme families are involved in HMOs metabolism. Specifically, to degrade HMOs, both β-1,4-galactosidases and two types of fucosidases, 1,2-α-l-fucosidase and 1,3-1,4-α-l-fucosidase, are needed. Fucosidases were also correlated to sialidases, which may act on both α-2,3 and α-2,6 linkages found in sialylated HMOs [2]. These results indicate that glycosidases that may potentially hydrolyze HMOs as well as glycans associated with mucin, sharing similar monomers, are strongly correlated and frequently found in the same MAG. Some HMOs-degrading enzymes were also strongly associated with the α-galactosidases characteristic of *B. bifidum*, confirming the glycolytic profiles elucidated for this species in previous sections. It should be noted that α-galactosidases have been reported only in specific bifidobacterial species [43] and may play a major in the metabolism of α-GOS like raffinose or stachyose, that are commonly added to infant formula [6].

## 4. Conclusions

A total of 1806 metagenome-assembled genomes (MAGs) corresponding to 177 different species have been recovered from 487 infant and adult metagenomes. Most MAGs recovered were taxonomically identified as *Escherichia coli*, *Faecalibacterium prausnitzii*, and *Ruminococcus gnavus*. Up to 203 MAGs have been recovered from the following *Bifidobacterium* species: *B. adolescentis*, *B. bifidum*, *B. breve*, *B. catenulatum*, *B. dentium*, *B. longum*, *B. pseudocatenulatum* and *B. scardovii*. Glycosidase activities annotation using the carbohydrate-active enzymes database (CAZy) coupled to mathematical modeling using machine-learning algorithms allowed elucidating characteristic glycosidase profiles for *B. adolescentis*, *B. bifidum*, *B. breve*, *B. longum* and *B. pseudocatenulatum* MAGs, showing high accuracy rates (>90%) when tested on an independent set of samples. GH5 44, GH32, and GH110 glycosidase families comprising hexosaminidases, fructosidases, fructosyl transferases and α-galactosidases were characteristic of *B. bifidum,* while β-galactosidases and hexosaminidases from GH1, GH2, GH5 and GH20 were characteristic of *B. adolescentis, B. breve* and *B. pseudocatenulatum*. Potential hydrolytic profiles of *B. longum* were characterized by the presence of GH1 and GH30 families involving β-galactosidases and fucosidases. Correlation networks of glycosidase activities revealed that glycosidases able to metabolize α- and β-GOS as well as HMOs were strongly correlated and are frequently present within the same MAG. The study of *Bifidobacterium* MAGs allows comparing metabolic profiles of species found in individuals following specific dietary interventions as well as assessing metabolic complementarities between bifidobacteria and non-bifidobacteria species found in the same participant. The data analysis strategy presented in this work is of particular interest to gain a better understanding of the carbohydrate metabolism of bifidobacteria, and this design could be easily translated to other health-promoting microorganisms in future works.

## Data Availability

Metagenome sequences from public repositories were used to conduct this study: infant metagenome dataset from Sequence Read Archive (SRA) deposited under BioProject identity PRJNA473126 (sample accession codes SAMN09259835–SAMN09260236) and adult metagenome dataset from MG-RAST deposited under MG-RAST identity mgp13068 (MG-RAST ID of sequences mgm4624578.3–mgm4624657.3).

## Supplementary Materials

The following are available online at https://www.mdpi.com/article/10.3390/microorganisms9051034/s1, Figure S1: Comparative account of different three algorithms used for classification of metagenome-assembled genomes (MAGs) of *B. adolescentis, B. bifidum, B. breve, B. longum* and *B. pseudocatenulatum* based on their glycosidase activities: random forest (RF), generalized linear model elastic-net (glmnet) and artificial neural network (ANN). Differences between models were calculated via their resampling distributions (number of resamples = 10). ^a^ No statistically significant (*p* > 0.05) differences between models were found; Table S1: Number of metagenome-assembled genomes (MAGs) recovered (n = 1806) per species and strains identified (n = 177); Table S2: Number of metagenome-assembled genomes (MAGs) recovered per diet type. GOS: galacto-oligosaccharides, FOS: fructo-oligosaccharides; Table S3: Glycosidase activities of interest included in selected CAZy families; Table S4: Sensitivity, specificity, precision, recall, F1 coefficients and balanced accuracy rates for artificial neural network (ANN) random forest (RF) and generalized linear model elastic-net (glmnet) computed to classify metagenome-assembled genomes (MAGs) of *B. adolescentis*, *B. bifidum*, *B. breve*, *B. longum* and *B. pseudocatenulatum* based on their glycosidase activities; Table S5: Variable importance coefficients of artificial neural network (ANN) computed to classify metagenome-assembled genomes (MAGs) of *B. adolescentis*, *B. bifidum*, *B. breve*, *B. longum* and *B. pseudocatenulatum* based on their glycosidase activities. Importance coefficients were determined by the sum of the product of raw input-hidden and hidden-output connection weights; Table S6: Variable importance coefficients of random forest (RF) computed to classify metagenome-assembled genomes (MAGs) of *B. adolescentis*, *B. bifidum*, *B. breve*, *B. longum* and *B. pseudocatenulatum* based on their glycosidase activities. Importance coefficients were determined by the permutation of the out-of-bag-error; Table S7: Variable importance coefficients of generalized linear model elastic-net (glmnet) computed to classify metagenome-assembled genomes (MAGs) of *B. adolescentis*, *B. bifidum*, *B. breve*, *B. longum* and *B. pseudocatenulatum* based on their glycosidase activities. Importance coefficients were determined by calculating the area under the ROC (receiver operating characteristic) curve.
