# Supplementary material for: A Machine Learning Approach to Study Glycosidase Activities from Bifidobacterium
Source: Microorganisms. 2021 May 11;9(5):1034. doi: 10.3390/microorganisms9051034 (PMC8151561; doi:10.3390/microorganisms9051034)
Supplement: Supplementary file 1 [file microorganisms-09-01034-s001.zip › microorganisms-1200062-supplementary.pdf]

Table S1. Number of metagenome-assembled genomes (**MAGs**) recovered (n=1806) per species and strains identified (n=177).

Taxonomic identification	Number of MAGs	Taxonomic identification	Number of MAGs
Escherichia coli	140	*Lachnospiraceae bacterium KM106-2*	7
Faecalibacterium prausnitzii	116	*Phascolarctobacterium faecium*	7
Ruminococcus gnavus	114	*Prevotella dentalis*	7
Collinsella aerofaciens	71	*Ruminococcus albus*	7
Bifidobacterium longum	70	*Alistipes onderdonkii*	6
Veillonella parvula	62	*Bacteroides vulgatus*	6
Akkermansia muciniphila	59	*Clostridium saccharolyticum*	6
Lachnospiraceae bacterium GAM79	50	*Haemophilus parainfluenzae*	6
Ruthenibacterium lactatiformans	46	*Lactococcus lactis*	6
Bifidobacterium bifidum	39	*Muribaculum sp. TLL-A4*	6
Bifidobacterium breve	38	*Roseburia hominis*	6
Blautia sp. SC05B48	38	*Veillonella rodentium*	6
Clostridium bolteae	36	*Alistipes communis*	5
Bifidobacterium pseudocatenulatum	35	*Alistipes shahii*	5
Faecalitalea cylindroides	35	*Bacteroides fragilis*	5
Ruminococcus sp. JE7A12	32	*Eggerthella lenta*	5
Parabacteroides distasonis	27	*Streptococcus thermophilus*	5
Ruminococcus bicirculans	26	*Turicibacter sp. H121*	5
Lachnoclostridium sp. YL32	25	*Absiella sp. 9CBEGH2*	4
Longibaculum sp. KGMB06250	25	*Barnesiella viscericola*	4
Absiella argi	23	*Butyrivibrio fibrisolvens*	4
Anaerostipes hadrus	22	*Clostridioides difficile*	4
Prevotella ruminicola	22	*Enterococcus avium*	4
Flavonifractor plautii	21	*Paraprevotella xylaniphila*	4
Lachnospira eligens	21	*Sutterella faecalis*	4
Lachnospiraceae bacterium	20	*Burkholderiales bacterium YL45*	3
Caproiciproducens sp. NJN-50	19	*Clostridium butyricum*	3
Veillonella atypica	19	*Clostridium cellulosi*	3
Veillonella dispar	18	*Dialister pneumosintes*	3
Bifidobacterium adolescentis	17	*Flintibacter sp. KGMB00164*	3
Clostridium sp. enoides	17	*Klebsiella aerogenes*	3
Ruminococcus champanellensis	17	*Lachnoclostridium phocaeense*	3
Blautia sp. N6H1-15	16	*Ligilactobacillus ruminis*	3
Lachnospiraceae bacterium Choco86	16	*Megamonas funiformis*	3
Enterococcus faecalis	15	*Methanobrevibacter smithii*	3
Erysipelotrichaceae bacterium GAM147	15	*Prevotella intermedia*	3
Clostridium perfringens	14	*Actinomyces pacaensis*	2
Megasphaera elsdenii	13	*Alistipes dispar*	2
Blautia producta	13	*Alistipes finegoldii*	2
Bacteroides uniformis	12	*Bacillus sp. SB49*	2
Klebsiella pneumoniae	12	*Bifidobacterium dentium*	2
Lacticaseibacillus rhamnosus	11	*Brachyspira pilosicoli*	2
Streptococcus sp. HSISS3	11	*Citrobacter freundii*	2
Roseburia intestinalis	9	*Citrobacter koseri*	2
Streptococcus salivarius	9	*Clostridium hylemonae*	2
Bacteroides thetaiotaomicron	8	*Clostridium scindens*	2
Enterococcus sp. HSIEG1	8	*Clostridium sp. CT4*	2
Oscillibacter valericigenes	8	*Enterobacter hormaechei*	2
Phascolarctobacterium succinatutens	8	*Eubacterium limosum*	2
Streptococcus lutetiensis	8	*Faecalibaculum rodentium*	2

Table S1. Cont.

Taxonomic identification	Number of MAGs	Taxonomic identification	Number of MAGs
Klebsiella michiganensis	2	*Lentilactobacillus hilgardii*	1
Klebsiella oxytoca	2	*Limosilactobacillus reuteri*	1
Muribaculaceae bacterium DSM	2	*Lactobacillus terrae*	1
Selenomonas sp. oral	2	*Leuconostoc lactis*	1
Serratia marcescens	2	*Mageeibacillus indolicus*	1
Staphylococcus epidermidis	2	*Megasphaera stantonii*	1
Succinivibrio dextrinosolvens	2	*Microbacterium aurum*	1
Weissella confusa	2	*Microbacterium hominis*	1
Acetobacterium woodii	1	*Neorhizobium galegae*	1
Actinomyces sp. oral	1	*Olsenella sp. GAM18*	1
Alistipes megaguti	1	*Paenibacillus sabinae*	1
Aminipila butyrica	1	*Prevotella denticola*	1
Anaerostipes rhamnosivorans	1	*Pseudobutyrivibrio xylanivorans*	1
Bacillus megaterium	1	*Rathayibacter sp. VKM*	1
Bacillus thuringiensis	1	*Rhizobium sp. NXC24*	1
Bacteroides coprosuis	1	*Serratia liquefaciens*	1
Bacteroides ovatus	1	*Streptococcus gallolyticus*	1
Bacteroides salanitronis	1	*Streptococcus sp. HSISM1*	1
Bacteroides xylanisolvens	1	*Streptococcus sp. HSISS2*	1
Bifidobacterium catenulatum	1	*Streptococcus sp. LPB0220*	1
Bifidobacterium scardovii	1	*Streptococcus vestibularis*	1
Bradyrhizobium erythrophlei	1	*Streptomyces qinzhouensis*	1
Brevibacillus agri	1	*Streptomyces sp. 769*	1
Burkholderia cenocepacia	1	*Streptomyces sp. S4.7*	1
Burkholderia multivorans	1	*Streptomyces sp. Sge12*	1
Butyrivibrio hungatei	1	*Sutterella megalosphaeroides*	1
Butyrivibrio proteoclasticus	1	*Virgibacillus phasianinus*	1
Cellulomonas sp. Z28	1		
Citrobacter portucalensis	1		
Citrobacter sp. CFNIH10	1		
Clostridiales bacterium CCNA10	1		
Clostridium botulinum	1		
Clostridium saccharobutylicum	1		
Clostridium sp. BNL1100	1		
Clostridium sp. JN-9	1		
Corynebacterium epidermidicanis	1		
Corynebacterium sp. 2184	1		
Corynebacterium stationis	1		
Cutibacterium avidum	1		
Dialister hominis	1		
Dialister massiliensis	1		
Eggerthella sp. YY7918	1		
Enterococcus casseliflavus	1		
Erysipelotrichaceae bacterium I46	1		
Ethanoligenens harbinense	1		
Finegoldia magna	1		
Intestinibaculum porci	1		
Klebsiella variicola	1		
Lachnospiraceae bacterium oral	1		
Companilactobacillus futsaii	1		

Table S2. Number of metagenome-assembled genomes (**MAGs**) recovered per diet type. **GOS**: galacto-oligosaccharides, **FOS**: fructo-oligosaccharides.

**Number of MAGs per diet type considering all species identified**	
**Taxonomic identification**	**Number of MAGs**
Infant formula-fed (infant)	638
Fiber-rich (adult)	467
Infant formula-fed + GOS (infant)	281
Breast-fed (infant)	144
Infant formula-fed + FOS (infant)	106
Infant formula-fed + GOS + FOS (infant)	84
Breast-fed + GOS (infant)	75
Whole-milk-fed (infant)	8
Breast-fed + GOS + FOS (infant)	3

Table S2. Cont.

Taxonomic identification	Total	*B. adolescentis*	*B. bifidum*	*B. breve*	*B. catenulatum*	*B. dentium*	*B. longum*	*B. pseudocatenulatum*	*B. scardovii*
	**Number of MAGs per diet type considering only *Bifidobacterium* species**								
Infant formula-fed (infant)	64	0	8	9	1	0	32	13	1
Infant formula-fed + GOS (infant)	52	2	5	16	0	0	20	9	0
Breast-fed (infant)	31	0	11	7	0	0	9	4	0
Infant formula-fed + GOS + FOS (infant)	17	5	7	0	0	2	2	1	0
Infant formula-fed + FOS (infant)	12	2	0	3	0	0	3	4	0
Fiber-rich (adult)	11	8	0	0	0	0	2	1	0
Breast-fed + GOS (infant)	11	0	5	3	0	0	2	1	0
Whole-milk-fed (infant)	4	0	2	0	0	0	0	2	0
Breast-fed + GOS + FOS (infant)	1	0	1	0	0	0	0	0	0

Table S3. Glycosidase activities of interest included in selected CAZy families.

CAZy family	Activities of interest included in the family
CBM32	Sialidase
CBM40	Sialidase
GH1	β-galactosidase
GH2	β-galactosidase
GH3	β-N-acetylhexosaminidase
GH4	α-galactosidase
GH5 1	β-N-acetylhexosaminidase
GH5 2	β-N-acetylhexosaminidase
GH5 4	β-N-acetylhexosaminidase
GH5 5	β-N-acetylhexosaminidase
GH5 7	β-N-acetylhexosaminidase
GH5 8	β-N-acetylhexosaminidase
GH5 10	β-N-acetylhexosaminidase
GH5 13	β-N-acetylhexosaminidase
GH5 18	β-N-acetylhexosaminidase
GH5 21	β-N-acetylhexosaminidase
GH5 22	β-N-acetylhexosaminidase
GH5 25	β-N-acetylhexosaminidase
GH5 37	β-N-acetylhexosaminidase
GH5 38	β-N-acetylhexosaminidase
GH5 41	β-N-acetylhexosaminidase
GH5 44	β-N-acetylhexosaminidase
GH5 46	β-N-acetylhexosaminidase
GH5 54	β-N-acetylhexosaminidase
GH16	endo-β-1,4-galactosidase
GH20	β-hexosaminidase
GH29	α-L-fucosidase ; α-1,3/1,4-L-fucosidase
GH30	β-fucosidase
GH30 1	β-fucosidase
GH30 2	β-fucosidase
GH30 3	β-fucosidase
GH30 4	β-fucosidase
GH30 5	β-fucosidase
GH30 6	β-fucosidase
GH30 8	β-fucosidase
GH30 9	β-fucosidase
GH31	α-galactosidase
GH32	invertase ; endo-inulinase ; β-2,6-fructan 6-levanbiohydrolase ; endo-levanase ; exo-inulinase ; fructan β--fructosidase/1-exohydrolase ; fructan β--fructosidase/6-exohydrolase ; sucrose:sucrose 1-fructosyltransferase ; fructan:fructan 1-fructosyltransferase ; sucrose:fructan 6-fructosyltransferase ; fructan:fructan 6G-fructosyltransferase ; levan fructosyltransferase ; [retaining] sucrose:sucrose 6-fructosyltransferase ; cycloinulo-oligosaccharide fructanotransferase.
GH33	sialidase ; trans-sialidase
GH35	β-galactosidase
GH36	α-galactosidase ; stachyose synthase ; raffinose synthase.
GH39	β-galactosidase.
GH42	β-galactosidase
GH58	endo-sialidase.
GH59	β-galactosidase
GH68	levansucrase ; β-fructofuranosidase ; inulosucrase
GH95	α-L-fucosidase ; α-1,2-L-fucosidase ; α-L-galactosidase
GH97	glucoamylase ; α-glucosidase ; α-galactosidase.
GH109	β-N-acetylhexosaminidase
GH110	α-galactosidase
GH139	α-2-O-Me-L-fucosidase.
GH141	α-L-fucosidase
GH147	β-galactosidase
GH151	α-L-fucosidase

Table S4. Sensitivity, specificity, precision, recall, F1 coefficients and balanced accuracy rates for artificial neural network (**ANN**) random forest (**RF**) and Generalized Linear Model Elastic-net (**GLMNET**) computed to classify metagenome-assembled genomes (**MAGs**) of *B. adolescentis, B. bifidum, B. breve, B. longum* and *B. pseudocatenulatum* based on their glycosidase activities.

**ANN**	**Sensitivity**	**Specificity**	**Precision**	**Recall**	**F1**	**Balanced accuracy**
Bifidobacterium adolescentis	0.63	1.00	1.00	0.63	0.77	0.81
Bifidobacterium bifidum	1.00	0.98	0.92	1.00	0.96	0.99
Bifidobacterium breve	1.00	0.98	0.91	1.00	0.95	0.99
Bifidobacterium longum	0.95	0.97	0.95	0.95	0.95	0.96
Bifidobacterium pseudocatenulatum	0.90	0.96	0.82	0.90	0.86	0.93

**RF**	**Sensitivity**	**Specificity**	**Precision**	**Recall**	**F1**	**Balanced accuracy**
Bifidobacterium adolescentis	0.71	1.00	1.00	0.71	0.83	0.86
Bifidobacterium bifidum	0.92	0.98	0.92	0.92	0.92	0.95
Bifidobacterium breve	1.00	0.98	0.91	1.00	0.95	0.99
Bifidobacterium longum	0.95	0.97	0.95	0.95	0.95	0.96
Bifidobacterium pseudocatenulatum	0.90	0.96	0.82	0.90	0.86	0.93

**GLMNET**	**Sensitivity**	**Specificity**	**Precision**	**Recall**	**F1**	**Balanced accuracy**
Bifidobacterium adolescentis	0.71	1.00	1.00	0.71	0.83	0.86
Bifidobacterium bifidum	0.92	0.98	0.92	0.92	0.92	0.95
Bifidobacterium breve	1.00	0.98	0.91	1.00	0.95	0.99
Bifidobacterium longum	0.95	0.97	0.95	0.95	0.95	0.96
Bifidobacterium pseudocatenulatum	0.90	0.96	0.82	0.90	0.86	0.93

Table S5. Variable importance coefficients of artificial neural network (**ANN**) computed to classify metagenome-assembled genomes (**MAGs**) of *B. adolescentis, B. bifidum, B. breve, B. longum* and *B. pseudocatenulatum* based on their glycosidase activities. Importance coefficients were determined by the sum of the product of raw input-hidden and hidden-output connection weights.

B. adolescentis	Importance (%)	*B. bifidum*	Importance (%)	*B. breve*	Importance (%)	*B. longum*	Importance (%)	*B. pseudocatenulatum*	Importance (%)
**GH2**	100.0	**GH110**	100.0	**GH5 18**	100.0	**GH30 5**	100.0	**GH5 44**	100.0
**GH30 2**	50.1	**GH20**	86.5	**GH1**	75.6	**GH5 18**	49.2	**GH20**	94.7
**GH32**	47.0	**CBM32**	82.3	**GH5 44**	29.6	**GH32**	22.3	**GH30 2**	60.7
**GH35**	26.3	**GH36**	57.6	**GH95**	10.0	**GH20**	20.2	**GH31**	31.5
**GH1**	21.3	**GH95**	51.7	**GH31**	9.4	**GH59**	12.9	**GH95**	10.4
**GH31**	17.7	**GH1**	34.1	**GH2**	7.9	**GH30 9**	12.9	**GH32**	6.0
**GH3**	15.5	**GH42**	27.9	**GH59**	3.0	**GH42**	8.2	**GH35**	3.8
**GH36**	15.1	**GH59**	16.0	**GH30 9**	3.0	**GH31**	5.8	**GH3**	2.1
**GH42**	11.6	**GH30 9**	16.0	**GH32**	1.0	**GH3**	0.3	**GH5 4**	0.0
**GH110**	8.8	**GH3**	10.7	**GH5 4**	0.0	**GH5 4**	0.0	**GH42**	-7.1
**GH5 44**	5.9	**GH35**	5.6	**GH36**	-3.4	**GH35**	-3.0	**GH1**	-10.0
**CBM32**	4.0	**GH5 4**	0.0	**CBM32**	-13.8	**GH36**	-4.5	**GH30 9**	-11.6
**GH5 4**	0.0	**GH30 5**	-12.3	**GH3**	-17.3	**GH95**	-4.5	**GH59**	-11.6
**GH30 5**	-19.1	**GH2**	-22.6	**GH35**	-18.1	**GH110**	-4.6	**CBM32**	-19.8
**GH59**	-31.1	**GH30 2**	-57.1	**GH20**	-22.9	**CBM32**	-7.5	**GH110**	-24.0
**GH30 9**	-31.1	**GH5 18**	-63.8	**GH110**	-26.1	**GH2**	-16.3	**GH36**	-30.4
**GH95**	-61.5	**GH31**	-117.1	**GH42**	-26.7	**GH30 2**	-16.8	**GH2**	-38.1
**GH5 18**	-67.9	**GH32**	-139.4	**GH30 2**	-33.5	**GH5 44**	-37.4	**GH30 5**	-100.5
**GH20**	-219.0	**GH5 44**	-139.8	**GH30 5**	-38.9	**GH1**	-64.9	**GH5 18**	-108.6

Table S6. Variable importance coefficients of random forest (**RF**) computed to classify metagenome-assembled genomes (**MAGs**) of *B. adolescentis, B. bifidum, B. breve, B. longum* and *B. pseudocatenulatum* based on their glycosidase activities. Importance coefficients were determined by the permutation of the out of-bag-error.

B. adolescentis	**Importance (%)**	***B. bifidum***	**Importance (%)**	***B. breve***	**Importance (%)**	***B. longum***	**Importance (%)**	***B. pseudocatenulatum***	**Importance (%)**
**GH20**	100.0	**GH32**	100.0	**GH5 18**	100.0	**GH30 5**	100.0	**GH5 18**	100.0
**GH5 18**	41.2	**GH5 44**	94.6	**GH1**	89.1	**GH1**	75.6	**GH30 5**	79.8
**GH30 5**	31.2	**GH5 18**	68.4	**GH30 5**	49.8	**GH5 18**	51.2	**GH5 44**	69.7
**GH110**	18.6	**GH31**	56.9	**GH95**	31.0	**GH110**	33.4	**GH20**	68.4
**GH95**	16.5	**GH30 5**	56.0	**GH110**	25.9	**GH32**	30.1	**GH110**	36.1
**GH2**	14.9	**GH20**	42.9	**GH5 44**	24.6	**GH95**	27.6	**GH30 2**	23.1
**GH32**	6.0	**GH110**	40.4	**GH32**	20.5	**GH30 2**	23.2	**GH31**	14.4
**CBM32**	4.2	**GH30 2**	26.9	**GH30 2**	14.4	**GH5 44**	22.6	**CBM32**	10.5
**GH31**	4.2	**GH35**	4.7	**GH2**	9.5	**GH20**	11.5	**GH36**	5.9
**GH35**	2.5	**CBM32**	2.7	**CBM32**	6.3	**GH36**	10.3	**GH42**	4.2
**GH3**	0.0	**GH1**	1.4	**GH35**	4.1	**GH35**	8.3	**GH30 9**	4.2
**GH5 4**	0.0	**GH5 4**	0.0	**GH5 4**	0.0	**CBM32**	6.8	**GH3**	0.0
**GH30 9**	0.0	**GH30 9**	0.0	**GH42**	0.0	**GH30 9**	5.2	**GH5 4**	0.0
**GH42**	0.0	**GH42**	0.0	**GH36**	-1.7	**GH2**	5.0	**GH59**	0.0
**GH59**	0.0	**GH59**	0.0	**GH3**	-2.1	**GH3**	3.3	**GH1**	-0.1
**GH5 44**	-0.4	**GH2**	-1.7	**GH20**	-2.2	**GH59**	2.3	**GH32**	-3.1
**GH36**	-0.5	**GH3**	-9.3	**GH31**	-7.4	**GH5 4**	0.0	**GH2**	-6.4
**GH30 2**	-3.9	**GH95**	-9.6	**GH30 9**	-8.8	**GH42**	0.0	**GH95**	-11.2
**GH1**	-9.3	**GH36**	-10.2	**GH59**	-9.2	**GH31**	-0.7	**GH35**	-16.8

Table S7. Variable importance coefficients of Generalized Linear Model Elastic-net (**GLMNET**) computed to classify metagenome-assembled genomes (**MAGs**) of *B. adolescentis, B. bifidum, B. breve, B. longum* and *B. pseudocatenulatum* based on their glycosidase activities. Importance coefficients were determined by calculating the area under the ROC (Receiver Operating Characteristic) curve.

B. adolescentis	Importance (%)	*B. bifidum*	Importance (%)	*B. breve*	Importance (%)	*B. longum*	Importance (%)	*B. pseudocatenulatum*	Importance (%)
**GH20**	100.0	**GH32**	100.0	**GH5 18**	100.0	**GH30 5**	100.0	**GH5 18**	100.0
**GH35**	28.4	**GH110**	84.0	**GH1**	70.1	**GH1**	53.4	**GH20**	96.8
**GH30 2**	25.2	**GH5 44**	74.2	**GH20**	22.0	**GH5 18**	44.0	**GH30 2**	74.1
**GH2**	23.0	**GH31**	65.6	**GH5 44**	18.5	**GH30 2**	25.6	**GH5 44**	73.7
**GH5 18**	18.6	**CBM32**	50.9	**GH95**	16.1	**GH5 44**	16.3	**GH30 5**	31.9
**GH95**	8.0	**GH42**	18.1	**GH30 2**	2.9	**GH2**	9.3	**GH36**	16.8
**CBM32**	0.0	**GH20**	8.0	**CBM32**	0.0	**GH30 9**	6.1	**GH31**	13.5
**GH1**	0.0	**GH1**	0.0	**GH2**	0.0	**GH59**	5.9	**GH2**	13.0
**GH3**	0.0	**GH2**	0.0	**GH3**	0.0	**GH20**	1.2	**CBM32**	0.0
**GH5 4**	0.0	**GH3**	0.0	**GH5 4**	0.0	**GH32**	1.0	**GH1**	0.0
**GH5 44**	0.0	**GH5 4**	0.0	**GH30 5**	0.0	**CBM32**	0.0	**GH3**	0.0
**GH30 5**	0.0	**GH5 18**	0.0	**GH30 9**	0.0	**GH3**	0.0	**GH5 4**	0.0
**GH30 9**	0.0	**GH30 2**	0.0	**GH31**	0.0	**GH5 4**	0.0	**GH30 9**	0.0
**GH31**	0.0	**GH30 5**	0.0	**GH32**	0.0	**GH31**	0.0	**GH32**	0.0
**GH32**	0.0	**GH30 9**	0.0	**GH35**	0.0	**GH35**	0.0	**GH35**	0.0
**GH36**	0.0	**GH35**	0.0	**GH36**	0.0	**GH36**	0.0	**GH42**	0.0
**GH42**	0.0	**GH36**	0.0	**GH42**	0.0	**GH42**	0.0	**GH59**	0.0
**GH59**	0.0	**GH59**	0.0	**GH59**	0.0	**GH95**	0.0	**GH95**	0.0
**GH110**	0.0	**GH95**	0.0	**GH110**	0.0	**GH110**	0.0	**GH110**	0.0

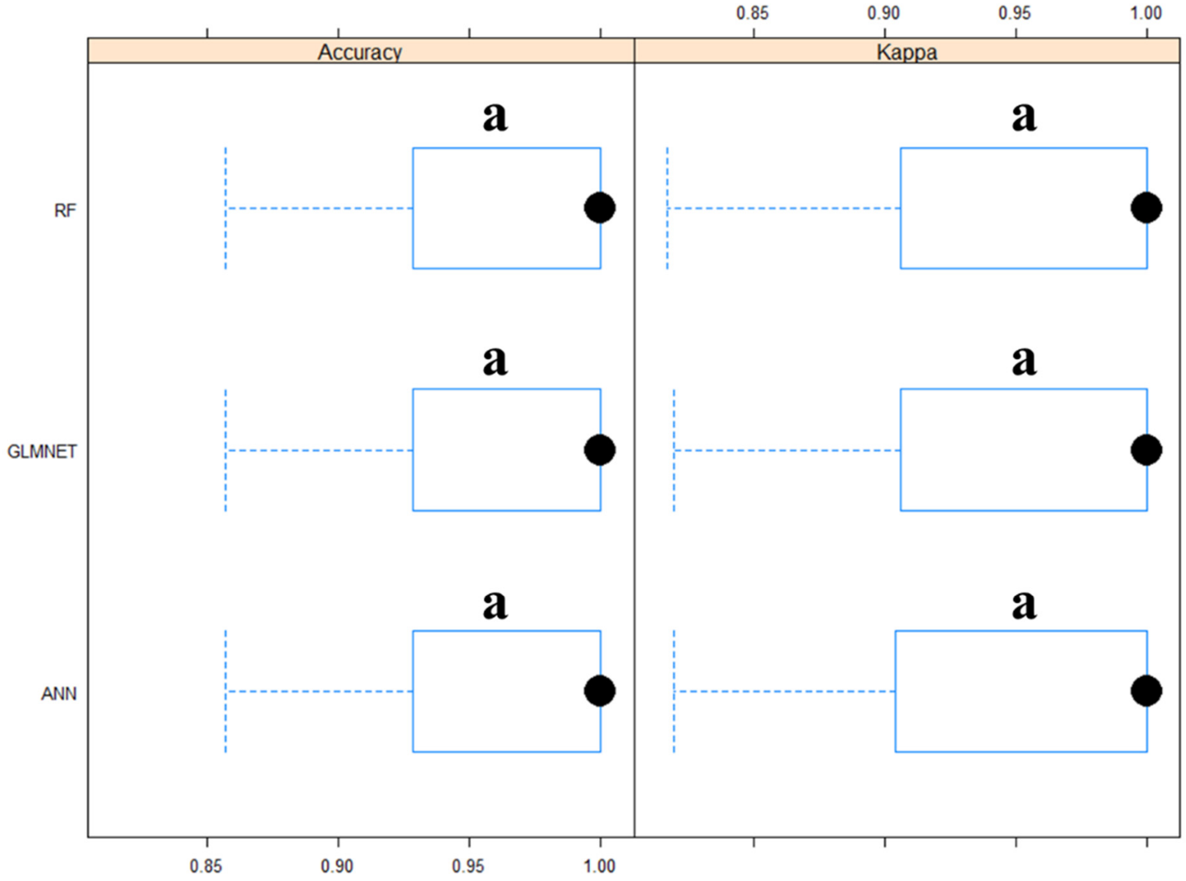

Figure S1. Comparative account of different three algorithms used for classification of metagenome-assembled genomes (**MAGs**) of *B. adolescentis, B. bifidum, B. breve, B. longum* and *B. pseudocatenulatum* based on their glycosidase activities: random forest (**RF**), Generalized Linear Model Elastic-net (**GLMNET**) and artificial neural network (**ANN**). Differences between models were calculated via their resampling distributions (number of resamples = 10). [a] No statistically significant ($p > 0.05$) differences between models were found.
